# Felt stigma and associated factors in children and adolescents with epilepsy: a multicenter cross-sectional study in China

**DOI:** 10.3389/fneur.2024.1459392

**Published:** 2024-08-14

**Authors:** Jing Zhao, Shuangzi Li, Ni Zhang, Cui Cui, Ting Wang, Mingping Fan, Junqi Zeng, Yuan Xie

**Affiliations:** ^1^Department of Nursing, Children’s Hospital of Chongqing Medical University, National Clinical Research Center for Child Health and Disorders, Ministry of Education Key Laboratory of Child Development and Disorders, Chongqing Key Laboratory of Pediatrics, Chongqing, China; ^2^Department of Neurology, Children’s Hospital of Chongqing Medical University, Chongqing, China; ^3^Department of Traditional Chinese Medicine, Children’s Hospital of Chongqing Medical University, Chongqing, China

**Keywords:** children and adolescents, epilepsy, stigma, social support, cross-sectional

## Abstract

**Objective:**

Epilepsy-related stigma is a global problem, yet there has been an inadequate focus on children and adolescents. The purpose of this study was to determine the status quo of stigma and its determinants among children and adolescents with epilepsy in China.

**Methods:**

A multicenter cross-sectional study was conducted across nine hospitals in eight cities within six provinces in China from 10 October 2023 to 15 June 2024. Participants included patients aged 8 to 18 years with epilepsy and their caregivers. Felt stigma was assessed with the Kilifi Stigma Scale for Epilepsy (KSSE). Social support and self-efficacy were collected through the Social Support Rating Scale (SSRS) and the Generalized Self-Efficacy Scale (GSES). The data were analyzed using t-tests, analysis of variance (ANOVA), Spearman correlation analysis, and multiple linear regression analysis.

**Results:**

The study enrolled 281 children and adolescents, with a mean age of 12.25 years (SD = 2.56), including 46.6% females. A total of 35.6% participants had self-reported felt stigma. The mean KSSE score is 9.58 (SD = 7.11). Meanwhile, stigma scores correlated strongly with reduced social support (r = −0.55, *p* < 0.01) and self-efficacy (r = −0.43, p < 0.01). Place of residence (rural vs. non-rural), academic performance (average and above vs. fair or poor), region (western region vs. non-western region), duration of epilepsy (≤5 years vs. >5 years), drug-resistant epilepsy (yes vs. no), comorbidities (yes vs. no), social support and self-efficacy are major influencing factors among the complex factors influencing the felt stigma among children and adolescents.

**Conclusion:**

Medical staff should be more aware of stigma among children and adolescents with epilepsy, especially those who live in rural and western areas, have poor academic performance, have epilepsy duration of more than 5 years, have drug-resistant epilepsy, and have comorbidities, who are at higher risk of stigma. It is recommended that effective measures be taken to alleviate stigma by improving children and adolescents’ self-efficacy and providing more social support for them and their families.

## 1 Introduction

Epilepsy is one of the most common neurological disorders that affects around 70 million people worldwide (1). Epilepsy has a high incidence in early childhood, with approximately 75% of epilepsy begins in childhood (2). It affects neurological, cognitive, psychological and social domains and accounts for more than 0.75% of the global burden of disease (3, 4). However, clinical indicators of epilepsy such as seizure frequency do not fully reflect the disease burden, the stigma can be more frustrating than the seizures themselves. The World Health Organization (WHO) has identified stigma as a significant contributor to poor physical and mental health in individuals with epilepsy (5). Epilepsy stigma can be conceptualized as both “felt” and “enacted.” “Enacted stigma” refers to the actual instances of discrimination based on the diagnosis of epilepsy; whereas “felt stigma” refers to the shame, embarrassment or disgrace associated with epilepsy or the fear of being discriminated against (6). Either form can strongly impact a child’s life. However, felt stigma may cause more personal anguish and unhappiness than enacted (5).

Epilepsy-related stigma is a global problem, especially in China (7). A survey of 20 Chinese cities found that 64.9% of adolescents and young adults with epilepsy, as well as 64.0% of parents or caregivers, chose not to disclose the condition to others, potentially indicating a stigma surrounding epilepsy (8). In a community-based survey, a third of people with epilepsy of all ages identified stigma as the most difficult part of living with epilepsy (9). In Uganda, the prevalence of high-perceived stigma among children and adolescents with epilepsy was 34.0% (10). Stigma can disrupt the psychosocial development in children with epilepsy, leading to increased anxiety, depression, and poor quality of life (11). Stigma and discrimination were found to impact negatively on healthcare, education, employment and income, which can have an economic impact (12).

Several factors have been identified as influencing the stigma associated with epilepsy, including gender, age, duration of epilepsy, seizure frequency, severity and type of epilepsy (13), place of residence (14), educational attainment (6), anti-seizure medication (ASMs) (15), self-efficacy and social support (16). In addition, a study in Bulgaria found that 43.6% of people with drug-resistant epilepsy felt stigmatized (17). Children with epilepsy experience poor academic performance and school dropout due to bullying, alienation and stigma (18). Social support is a protective factor of subjective well-being. Individuals with good social support usually have an increased sense of control, which helps them cope better with adversity (16). Previous research shows that stigma is negatively associated with self-efficacy among adult with epilepsy (19). Successful epilepsy management, which necessitates strong social support and self-efficacy, is essential for individuals to regain their roles in school and social life, thereby enhancing their overall well-being (19).

Successful integration of children and adolescents with epilepsy into school and society is important goal in epilepsy care (20). The ability to overcome stigma is important determinants of successful integration. Yet the effect of stigma and its associated factors on children and adolescents populations are poorly understood. Therefore, the aim of this study is to assess the extent of felt stigma and its associated factors to fill this research gap. Furthermore, interventions aimed at reducing stigma and improving subjective well-being can be developed based on these findings.

## 2 Materials and methods

### 2.1 Study design and population

A multicenter cross-sectional survey was conducted in China, covering nine tertiary children’s and comprehensive hospitals in different regions of China from 10 October 2023 to 15 June 2024. The managers of the neurology units in the survey gave their consent before the survey. All patients who were receiving neurologic services in the neurology outpatient or inpatients comprised the study population. The inclusion criteria were as follows: (i) the children and adolescents ranged in age from 8–18 years and were diagnosed according to 2017 classification and terminology of the International League Against Epilepsy (ILAE) for 2 months or longer (21); (ii) caregivers older than 18 years who provided care for children and adolescents with epilepsy, only one of the caregivers who was present with the child during the period of data collection was included in the study; (iii) both child and caregiver were able to read and understand the questionnaire independently or with the help of the researcher; (iv) they provided informed consent and voluntarily participated in this study. Exclusion criteria are as follows: (i) children and adolescents with intellectual impairment, and IQ score lower than 80 on the Wechsler Intelligence Scale were identified via medical records and caregiver consultations; (ii) caregivers were diagnosed with severe medical conditions, cognitive impairment, or mental illness.

### 2.2 Instrument

#### 2.2.1 Demographic characteristics

Using questionnaires, we collected general demographic data, including children’s gender, age, height, weight, academic performance (standardized test scores and class ranks, normalized by class size), basic family information (e.g., only child status), and region of residence (based the National Bureau of Statistics of China). Socioeconomic indicators such as guardian education level, household income level based on the 2022 National Bureau of Statistics, and medical insurance status were included. Notably, China’s medical insurance system includes both social and commercial insurance schemes. The actual coverage rate is over 80% for inpatient medical expenses for pediatric patients. We also assessed family upbringing styles: authoritative (high expectations, supportive, promoting independence), permissive (nurturing, lenient, with minimal control), neglectful (low engagement, guidance lacking), and autocratic (strict, demanding, with punitive measures).

#### 2.2.2 Disease-related characteristics

Disease-related characteristics involved epilepsy duration, the number of antiseizure medication (ASMs) types taken, seizure type, whether or not the diagnosis was drug resistant epilepsy (DRE) by neurologists (referring to the ILAE definition of DRE as “inadequate response to two appropriately chosen and used ASMs schedules”) (22), and seizure frequency in recent 3 months. These were mainly collected by medical records and self-report of caregivers.

#### 2.2.3 Stigma

The Kilifi Stigma Scale for Epilepsy (KSSE) is a three-point Likert 15-item scoring scale (23), which was used to measure felt stigma. The Chinese version of KSSE were translated and validated by Song et al. (24). The translated version exhibits good reliability and validity (Cronbach’s α = 0.93). The KSSE has been extensively validated in children and adolescents populations and has demonstrated cultural relevance and sensitivity in China (25). The total score ranges from 0 to 30. A score above the 66th percentile on the total scale is indicative of felt stigma, whereas a score below this threshold shows the absence of stigma.

#### 2.2.4 Social support

The Social Support Rating Scale (SSRS) was used to measure the degree of support received from friends, relatives, and healthcare providers, which was developed by a Chinese researcher (26). It comprises 10 items designed to evaluate objective support (3 items), subjective support (4 items), and utilization of support (3 items). Objective support reflects an individual’s social network, material direct support, and emotional support. Subjective support refers to the feeling of being respected, supported, and understood. Utilization of support reflects the respondents to seek and use the degree of social support. The scale had good predictive validity and internal consistency among Chinese population [Cronbach’s α = 0.82, (27)]. The overall score ranges from 12 to 66. Higher scores indicate stronger social support.

#### 2.2.5 Self-efficacy

The Generalized Self-Efficacy Scale (GSES) is a psychometric tool used to measure the level of generalized self-efficacy, developed by Jerusalem and Schwartz in 1979 (28). Self-efficacy is an individual’s belief in their ability to successfully manage different life situations (29). The Chinese version of GSES were translated and validated by Wang et al. (30). GSES has been translated into more than 28 languages and demonstrated strong reliability and validity (Cronbach’s α = 0.88) (31). The items are rated on a four-point scale, and possible total scores range from 10 to 40, with lower scores indicating lower self-efficacy.

### 2.3 Ethical statement

Participation in this study was completely voluntary and anonymous. Before completing the questionnaire, the researchers obtained informed consent from the participants and their caregivers. This study was approved by the Medical Ethical Committee of Children’s Hospital of Chongqing Medical University (No. 2023-370).

### 2.4 Data collection methods

Prior training was provided to all researchers working in a clinical setting. The questionnaire was distributed to neurology outpatient or inpatients. The participants were recruited using a convenient sampling method. Given the sensitivity of the stigma issue, measures were taken to ensure data authenticity. The researchers collected the information through a face-to-face interview, by providing participants with consistent instructions, instructions for completing the questionnaire, and links to access the questionnaire. Researchers obtained their informed consent before completing the questionnaire. For children and adolescents, consent was obtained from the legal guardian. Participants were informed that all data would be presented in statistical form to ensure anonymity and confidentiality. At the end of the survey, the data were checked for validity and completeness. If the questionnaire was incomplete, the respondent was asked to complete the missing items immediately. A total of eight cities in six provinces were surveyed in China, including the eastern, central and western regions. The division of regions was provided by the National Bureau of Statistics of China website. Finally, 302 children and adolescents with epilepsy and caregivers were recruited and completed the survey. A total of 281 valid questionnaires were recovered, with a 93.0% valid return rate.

### 2.5 Statistical analysis

We used SPSS 26.0 to accomplish all the statistical analyses. Demographic data and disease-related characteristics were summarized as means (standard deviations) and as frequency counts (percentages) for categorical variables. Comparisons of KSSE scores with demographic and clinical variables were analyzed using independent t-tests or one-way analysis of variance (ANOVA). Post-hoc comparisons for significant variables were performed using the Bonferroni correction. Correlation between stigma scores, epilepsy duration, social support, and self-efficacy using Spearman correlation. Multiple linear regression analyses of statistically significant independent variables were performed to derive factors influencing stigma in children and adolescents with epilepsy. Academic performance was coded as dummy variables. The results are reported as unstandardized (B) and standardized (β) regression coefficients, and the R-squared was reported for each outcome variable. All tests were two-sided with a significance level of *p* < 0.05.

## 3 Results

### 3.1 Participants and characteristics

A total of 281 children and adolescents with epilepsy and their caregivers were included. All patients’ demographic characteristics have been presented in Table 1. The mean age of children and adolescents with epilepsy was 12.25 years (SD = 2.56). One hundred and thirty one (46.6%) of them were female, and 88 patients (31.3%) lived in rural areas. Most families (87.9%) have medical insurance. One hundred and thirteen patients (40.2%) had a monthly household income of more than 5, 000 yuan. Only 32 mothers (11.4%) had attended university or above, while nearly 19.2% of fathers had attended university or above. One hundred and thirty-seven (48.7%) had below average academic performance. Table 2 shows the disease-related characteristics of patients. The mean duration of epilepsy since diagnosis was 3.52 years (SD = 2.95), and 113 (40.2%) took more than one type of ASMs. 148 (52.7%) of seizure type had focal onset. 20.3% of patients reported were drug-resistant epilepsy. Focal onset seizures were reported in 148 (52.7%) patients, and 20.3% of patients were diagnosed with drug-resistant epilepsy. Seizure freedom during the recent 3 months was noted in 100 (35.6%) patients, and 143 patients (50.9%) reported had comorbidities.

**Table 1:** Demographic characteristics of study patients.

Variables	Classification	Number (%) or mean ± SD	KSSE		
			Mean ± SD	t or F	*P*-value
Age (years)		12.25 ± 2.56			
	8–11	129 (45.9%)	10.06 ± 7.17	0.82	0.44
	12–14	105 (37.4%)	8.89 ± 6.92		
	15–18	47 (16.7%)	9.83 ± 7.41		
Gender	Male	150 (53.4%)	9.42 ± 7.02	−0.41	0.68
	Female	131 (46.6%)	9.77 ± 7.24		
Place of residence	Urban	84 (29.9%)	8.37 ± 6.04^a^	8.77	**<0.01**
	Town	109 (38.8%)	9.10 ± 7.09^a^		
	Rural	88 (31.3%)	11.34 ± 7.80^b^		
Medical insurance	Yes	247 (87.9%)	9.49 ± 7.07	−0.57	0.57
	No	34 (12.1%)	10.24 ± 7.52		
Average monthly household income (CNY)	<3, 000	68 (24.2%)	11.76 ± 8.14^a^	3.03	**0.03**
	3, 000–5, 000	100 (35.6%)	9.22 ± 6.63^b^		
	5, 001–10, 000	75 (26.7%)	8.49 ± 6.78^b^		
	>10, 000	38 (13.5%)	8.79 ± 6.43^b^		
Medical expenses payment	Difficult to pay	81 (28.8%)	12.31 ± 7.96	3.85	**<0.01**
	Able to pay	200 (71.2%)	8.48 ± 6.44		
Mother’s education	Junior high school or below	120 (42.7%)	11.72 ± 7.83^a^	10.56	**<0.01**
	Senior high school or junior college	129 (45.9%)	8.26 ± 6.20^b^		
	University or above	32 (11.4%)	6.91 ± 5.57^b^		
Father’s education	Junior high school or below	107 (38.1%)	10.68 ± 6.99^a^	3.62	**0.03**
	Senior high school or junior college	120 (42.7%)	9.53 ± 7.36^a^		
	University or above	54 (19.2%)	7.52 ± 6.43^b^		
		20.07 ± 2.84			
BMI (kg/m^2^)	<18.5	79 (28.1%)	9.22 ± 7.38	0.69	0.50
	18.5–23.9	184 (65.5%)	9.89 ± 7.14		
	≥24	18 (6.4%)	8.06 ± 5.61		
Guardians	Parents	208 (74%)	9.15 ± 6.96	1.51	0.22
	Grandparents	64 (22.8%)	10.86 ± 7.73		
	Others	9 (3.2%)	10.56 ± 5.22		
One-child family	Yes	72 (25.6%)	9.67 ± 7.28	0.12	0.91
	No	209 (74.4%)	9.56 ± 7.07		
Academic performance	Excellent	20 (7.1%)	6.25 ± 6.24^a^	4.36	**<0.01**
	Good	42 (14.9%)	7.74 ± 6.76^a^		
	Average	71 (25.3%)	8.08 ± 6.00^ac^		
	Fair	74 (26.3%)	10.50 ± 6.72^bc^		
	Poor	63 (22.4%)	12.00 ± 8.10^bc^		
	Missing	11 (3.9%)	12.36 ± 8.02^c^		
Household type	Two-parent	109 (38.8%)	9.77 ± 7.45	1.25	0.29
	Multi-generational	129 (45.9%)	9.35 ± 6.66		
	Single parent	16 (5.7%)	8.26 ± 6.61		
Family upbringing	Authoritative	128 (45.6%)	8.46 ± 6.77	2.16	0.09
	Permissive	70 (24.9%)	10.87 ± 7.33		
	Neglectful	44 (15.7%)	9.86 ± 7.34		
	Autocratic	39 (13.9%)	10.64 ± 7.28		
Region	Western region	185 (65.8%)	10.39 ± 7.66^a^	3.93	**0.02**
	Eastern region	64 (22.8%)	7.89 ± 5.33^b^		
	Central region	32 (11.4%)	8.28 ± 6.31^b^		

KSSE, The Kilifi Stigma Scale for Epilepsy; SD, Standard Deviation; BMI, body mass index; CNY, Chinese yuan. The results of the post-hoc analysis (Bonferroni) were indicated by the letters a, b, and c in the table. Different letters indicated a statistical difference (*p* < 0.05), while the same letter indicated no significant difference (*p* > 0.05). Bold values indicate statistical significance.

**Table 2:** Disease-related characteristics of study patients.

Variables	Classification	Number (%) or mean ± SD	KSSE		
			Mean ± SD	t or F	*P*-value
Duration of epilepsy		3.52 ± 2.95			
	<1 years	60 (21.8%)	7.25 ± 5.50^a^	10.76	**<0.01**
	1–5 years	143 (52%)	9.08 ± 6.54^a^		
	>5 years	72 (26.2%)	12.63 ± 8.50^b^		
The number of ASMs types taken	0	24 (8.5%)	8.5 ± 6.64	0.48	0.69
	1	144 (51.2%)	9.94 ± 7.44		
	2	82 (29.2%)	9.63 ± 7.24		
	≥3	31 (11.0%)	8.65 ± 5.54		
Seizure type	Focal onset	148 (52.7%)	9.58 ± 7.14	0.63	0.60
	Generalized onset	84 (29.9%)	9.6 ± 7.36		
	Mixed	24 (8.5%)	11 ± 7.64		
	Unknown or unclassified	25 (8.9%)	8.2 ± 5.60		
Drug-resistant epilepsy	Yes	57 (20.3%)	13.84 ± 7.98	4.68	**<0.01**
	No	224 (79.7%)	8.5 ± 6.46		
Seizure frequency in past 3 months	0	100 (35.6%)	9.01 ± 6.79^a^	3.27	0.02
	1–3	126 (44.8%)	8.88 ± 6.73^a^		
	4–12	35 (12.5%)	12.09 ± 7.78^b^		
	>12	20 (7.1%)	12.5 ± 8.58^b^		
Length of seizures	<30s	67 (23.8%)	8.94 ± 7.09	0.68	0.57
	30 s–1 min	120 (42.7%)	10.23 ± 7.42		
	1–3 min	61 (21.7%)	9.48 ± 6.46		
	>3 min	33 (11.7%)	8.73 ± 7.26		
Loss of consciousness during seizure	Yes	239 (85.1%)	9.63 ± 7.27	0.27	0.79
	No	42 (14.9%)	9.31 ± 6.19		
Comorbidities	Yes	138 (49.1%)	12.25 ± 7.88	6.59	**<0.01**
	No	143 (50.9%)	7.01 ± 5.13		

KSSE, The Kilifi Stigma Scale for Epilepsy; SD, Standard Deviation; ASMs, antiseizure medication. The results of the post-hoc analysis (Bonferroni) were indicated by the letters a, b, and c in Table 1. Different letters indicated a statistical difference (*p* < 0.05), while the same letter indicated no significant difference (*p* > 0.05). Bold values indicate statistical significance.

### 3.2 The status quo and univariate analysis of stigma

**Figure 1:**
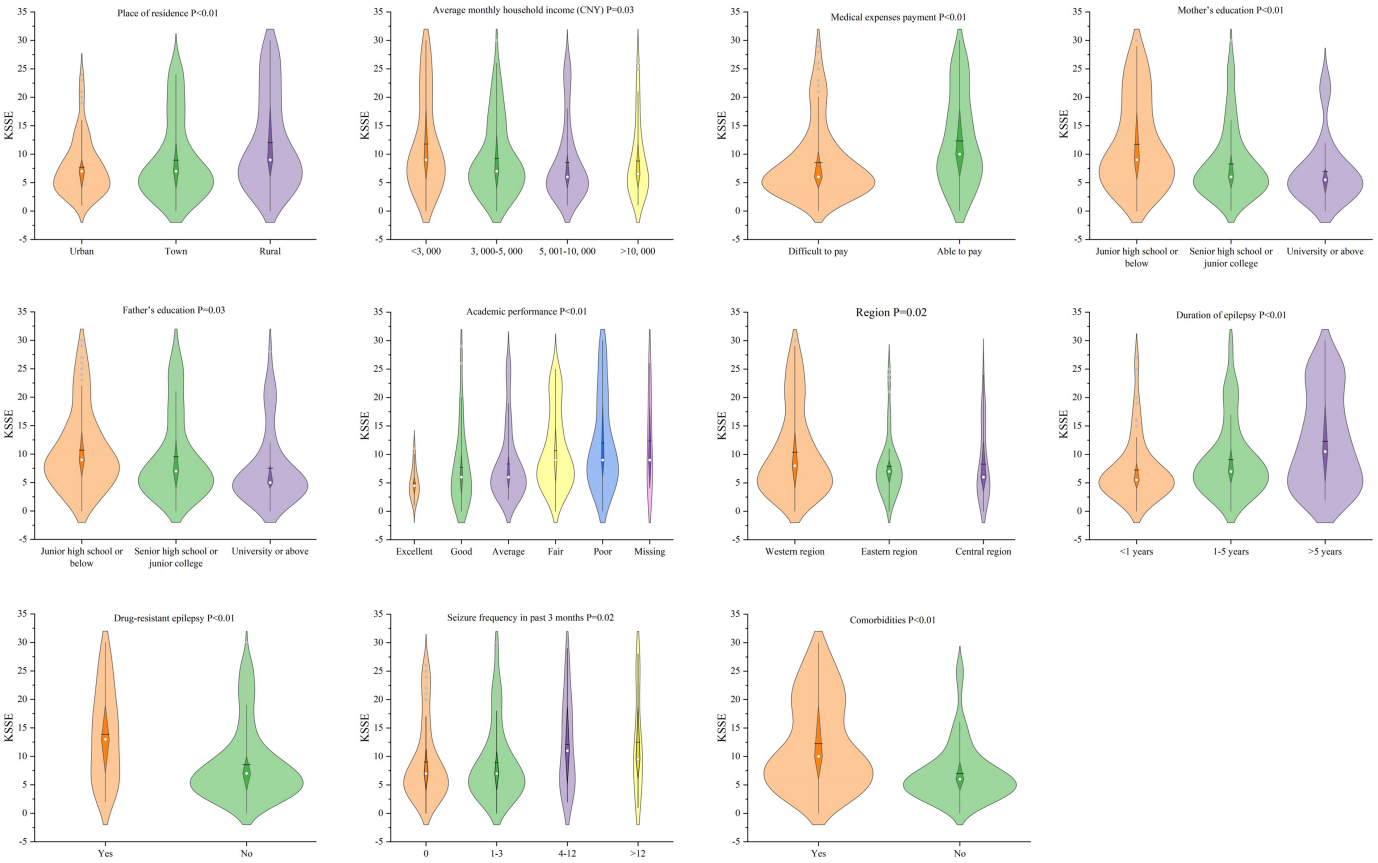
Violin plots of KSSE score in each group.

The mean KSSE score of 281 patients is 9.58 (SD = 7.11). Classification of the sample using the KSSE cut-off points revealed that 35.6% were at the threshold of feeling stigmatized. Through analysis, place of residence, monthly household income, difficulty of paying medical expenses, degree of mother’s and father’s education, children’s academic performance, family residence region, duration of epilepsy, drug-resistant epilepsy, seizure frequency in past 3 months, comorbidities have statistical significance (*p* < 0.05) with the stigma of children and adolescents in China, whereas different ages, gender, household type and the seizure type display no significant differences (*p* > 0.05). Details of post-hoc pairwise comparisons are shown in Tables 1, 2 and Figure 1.

### 3.3 Correlation analysis of stigma with social support and self-efficacy

**Figure 2:**
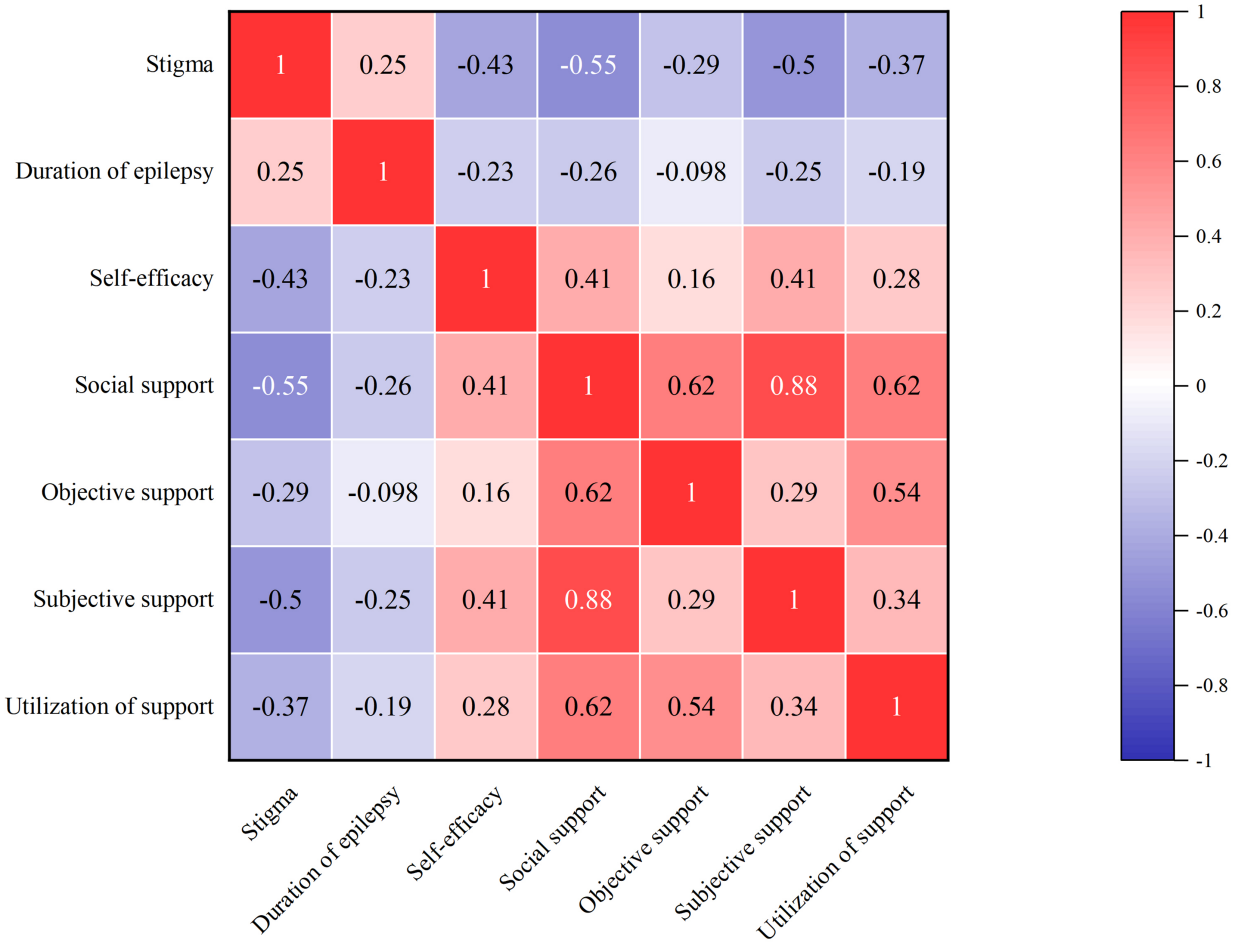
The correlation analysis of stigma, social support and self-efficacy.

The average social support score was 37.80 (SD = 9.25), and the score of subjective support dimension was the highest 21.94 (SD = 7.35). The self-efficacy score in children and adolescents with epilepsy was 26.77 (SD = 4.58). The result of the correlation analysis is shown in Figure 2. These analyses revealed stigma to be negatively correlated with social support (r = −0.55, *p* < 0.01) and self-efficacy (r = −0.43, *p* < 0.01).

### 3.4 Multiple linear regression analysis of stigma

The results of the variance analysis indicated that the discrepancy in stigma scores was primarily between rural and non-rural areas. Therefore, the place of residence was divided into rural and non-rural categories, This was done for other variables, like income, education, and so on. Factors with statistical significance in the univariate analysis and correlation analysis were regarded as independent variables; the KSSE scale score was taken as a dependent variable. The results of multiple linear regression on the KSSE are shown in Table 3. According to regression results, major influencing factors are comprised of place of residence, academic performance, region, duration of epilepsy, drug-resistant epilepsy, comorbidities, social support and self-efficacy, explaining 47.9% of the total variation in stigma scores among children and adolescents with epilepsy.

**Table 3:** Multiple linear regression for KSSE.

Variable	B	SE	Beta	t	*p*
Constant	37.43	2.65		14.11	0.00
Place of residence					
Rural vs. Non-rural	−1.37	0.69	−0.09	−1.99	**0.05**
Average monthly household income (CNY)					
<3, 000 vs. ≥3,000	−0.11	0.82	−0.01	−0.14	0.89
Medical expenses payment					
Difficult to pay vs. Able to pay	−1.13	0.77	−0.07	−1.48	0.14
Mother’ education					
Junior high school or below vs. Senior high school or junior college or above	−0.89	0.71	−0.06	−1.25	0.21
Father’ education					
Senior high school or junior college or below vs. University or above	0.48	0.86	0.03	0.56	0.58
Academic performance					
Average or above vs. Fair or Poor	1.63	0.65	0.12	2.52	**0.01**
Average or above vs. Missing	1.85	1.68	0.05	1.10	0.27
Region					
Western region vs. Non-western region	−1.65	0.67	−0.11	−2.47	**0.01**
Duration of epilepsy					
≤5 years vs. >5 years	1.86	0.74	0.11	2.51	**<0.01**
Drug-resistant epilepsy					
Yes vs. No	−1.61	0.84	−0.09	−1.91	**0.06**
Seizure frequency in recent 3 months					
≤3 vs. >3	0.33	0.83	0.02	0.40	0.69
Comorbidities					
Yes vs. No	−2.60	0.66	−0.18	−3.96	**<0.01**
SSRS	−0.22	0.04	−0.29	−5.76	**<0.01**
GSES	−0.38	0.08	−0.24	−4.90	**<0.01**

B, regression coefficient; SE, standard error; Beta, the standardized partial regression coefficient. SSRS, the Social Support Rating Scale; GSES, the Generalized Self-Efficacy Scale; Adjusted R^2^ = 0.479; F = 40.94; *p* < 0.01. Bold values indicate statistical significance.

## 4 Discussion

The present study utilized a multicenter cross-sectional design to investigate the degree of felt stigma and the influencing factors among children and adolescents with epilepsy in China. Results showed that 35.6% children and adolescents with epilepsy had felt stigma, which is higher than adults with epilepsy in China (32, 33), but significantly lower than the reported 44.7% in Ethiopia (34). Furthermore, this finding is closely similar to that of children and adolescents with epilepsy in Uganda (34.0%) (10). Differences in the incidence of stigma may be due to a complex array of sociocultural factors. The vulnerability to stigma during childhood and adolescence stems from underdeveloped coping mechanisms and limited social resources, making young individuals more prone to the effects of stigma compared to adults (12). Further research is needed to develop appropriate interventions to reduce stigma and improve health outcomes for children and adolescents.

Various factors may contribute to the stigma of epilepsy. The place of residence of the children and adolescents was found to have a statistically significant association with the level of perceived stigma. This finding is similar to the studies in Ethiopia that revealed that stigma is more prevalent among people with epilepsy in rural areas than in urban areas (35). People in rural areas with less education and lower socioeconomic status tend to have more negative attitudes toward epilepsy (36). Moreover, there is a huge treatment gap between urban and rural areas in China (1) This vast treatment gap is mainly driven by deficiencies in health care resources and the social stigma of epilepsy that is a result of cultural beliefs. The adverse effects of stigma on health conditions perpetuate a vicious cycle of social marginalization and deteriorating health (37).

The underdeveloped region of western China has a relatively weak social and economic foundation, resulting in a low level of social security (38). There is an uneven distribution of neurologists and healthcare resources between regions. In less developed areas, medical resources are scarce, especially in central and western China (38). This study showed that the western and non-western regions were statistically significant in the level of felt stigma. However, there is limited research on regional disparities in the stigma associated with epilepsy in China, and future studies with larger, multicenter samples are necessary for validation.

This study found that 48.7% of children with epilepsy performed below the average academic level, and this poor performance was linked to higher stigma. A Swedish study has also found that academic difficulties were common in schoolchildren with active epilepsy (39). A study that examined the Intelligence Quotient (IQ) screening and academic achievement testing of 173 children with epilepsy found that 48% of children had displayed learning disabilities (40). The stigma associated with epilepsy can negatively affect education, employment, income, and public opinion about resource allocation (41). The results indicate the need for intensive support and social care for children with epilepsy in educational settings.

The results of the study showed that children and adolescents with epilepsy for more than 5 years were more likely to feel stigmatized than those with epilepsy for less than 5 year, which is consistent with the previous studies (42). The chronicity of epilepsy may affect the child’s academic and social activities, leading to a wider gap with their peers, and this gap and isolation may exacerbate feelings of stigmatization (12). Children and adolescents undergo a critical developmental period that heightens their awareness of their illness and social status. The chronic and unpredictable nature of epilepsy can exacerbate their self-perception, leading to increased feelings of frustration and shame, and significantly affect emotional and psychological well-being (43). Therefore, appropriate education and advice should be provided to children and adolescents with a long duration of epilepsy to improve their self-management ability and establish correct disease perceptions.

Drug-resistant epilepsy means that conventional anti-seizure medication (ASMs) cannot be effectively control seizures, and children may need to take multiple medications. Frequent seizures and long-term treatment process are prone to psychiatric comorbidities such as anxiety and depression (44). The complexity of the disease can exacerbate the stigma for children and adolescents. In a study of patients hospitalized for epilepsy in Hong Kong, more than half had physical or psychiatric comorbidities (45). It is clear that the double stigma associated with epilepsy and its comorbidities has a negative impact on prevention and care efforts (46). Double stigma poses a new challenge, making it even more important to invest energy and resources. Further robust data collection and monitoring to identify intervention points is urgently needed.

We found that felt stigma was negatively related to a level of self-efficacy and social support. DiIorio et al. (47) identified that self-management interventions aimed at improving self-efficacy can reduce stigma in people with epilepsy. Children and adolescents with epilepsy who possess high levels of self-efficacy often have better emotional regulation skills, which in turn makes them more likely to experience satisfaction and positive emotions (48). Furthermore, the labeling theory proposed by Link could help us further explain this result, suggesting that stigma impairs mental health by destroying the self-evaluation dimension, a concept which is mainly related to self-efficacy (49). At a family level, higher levels of social support in various forms may be a protective factor against stigma. Previous studies have indicated that social support was inversely associated with felt stigma (27, 50). Therefore, increasing social support may be an intervention strategy to prevent or reduce felt stigma among children and adolescents with epilepsy.

### 4.1 Strengths, limitations, and future research directions

To our knowledge, this multicenter study is the first to examine multiple social determinants (parental education, region, academic performance, etc.) and disease characteristics (drug-resistant epilepsy, seizure frequency in past 3 months, comorbidities etc.) simultaneously in a population sample of children and adolescents with epilepsy. The results may provide the basis and direction for the prevention of stigma in children and adolescents with epilepsy. The study also has limitations. Firstly, it is a cross-sectional study, so causality cannot be determined between the variables. Follow-up researchers can conduct longitudinal studies to accumulate evidence of the causal relationship between felt stigma and various influencing factors. Secondly, although our sample size was multicenter, samples from the northeastern region of China were missing, which includes Heilongjiang, Jilin, and Liaoning provinces. This regional bias may affect the generalizability of our results. In future research, a more representative sample of all the regions in China will be needed to verify the results of this study. Thirdly, the traditional Chinese cultural and folk traditions, which influence the perception of epilepsy, may restrict the applicability of our findings in other cultural contexts. For example, the interpretation of epilepsy in Traditional Chinese Medicine, associating it with insanity and convulsion (7), could influence global understanding and research of the condition. Finally, other variables such as psychosocial issues, parental attitudes, school factors, and public attitudes and behaviors will be introduced into the research to discuss their impact on felt stigma in the future (51, 52).

## 5 Conclusion

This study indicates that stigma is a significant issue among children and adolescents with epilepsy in China. Meanwhile, place of residence, academic performance, region, duration of epilepsy, drug-resistant epilepsy, comorbidities, social support and self-efficacy are major influencing factors among the complex factors influencing the felt stigma among children and adolescents. It is crucial to integrate psychological counseling into clinical care, collaborate with schools to enhance academic support and reduce bullying, and engage policymakers to improve legislation and health insurance policies, particularly to extend coverage for epilepsy treatment in rural areas. These measures aim to mitigate the stigma and provide a more supportive environment for children and adolescents with epilepsy.

## Data Availability

The original contributions presented in the study are included in the article/supplementary material, further inquiries can be directed to the corresponding authors.
